# Oviposition Deterrent and Larvicidal Activity of *Salvia munzii* Essential Oil Against Susceptible and Insecticide-Resistant *Aedes aegypti*

**DOI:** 10.3390/tropicalmed11050134

**Published:** 2026-05-15

**Authors:** Selene M. Gutierrez-Rodriguez, Ivan Cordova-Guerreo, Laura Diaz-Rubio, Jesus A. Davila-Barboza, Iram P. Rodriguez-Sanchez, Beatriz Lopez-Monroy, Sergio A. Galindo-Rodriguez, Adriana E. Flores

**Affiliations:** 1 Facultad de Ciencias Biologicas, Universidad Autonoma de Nuevo Leon, Av. Universidad s/n Cd. Universita, San Nicolas de los Garza 66455, N.L., Mexico; selene.gutierrezrdr@uanl.edu.mx (S.M.G.-R.); jdavilab@uanl.edu.mx (J.A.D.-B.); iram.rodriguezsa@uanl.edu.mx (I.P.R.-S.); beatriz.lopezmr@uanl.edu.mx (B.L.-M.);; 2 Facultad de Ciencias Quimicas en Ingeniería, Universidad Autonoma de Baja California, Calzada Universidad 14418, Tijuana 22390, B.C., Mexico; icordova@uabc.edu.mx (I.C.-G.); ldiaz26@uabc.edu.mx (L.D.-R.)

**Keywords:** *Aedes aegypti*, essential oil, *Salvia munzii*, oviposition deterrence, larvicidal activity, insecticide resistance, vector control

## Abstract

The increasing prevalence of insecticide resistance in *Aedes aegypti* threatens the effectiveness of chemical vector control and highlights the need for alternative approaches targeting mosquito behavior. This study evaluated the oviposition deterrent and larvicidal activity of *Salvia munzii* essential oil against insecticide-susceptible (New Orleans; NO) and insecticide-resistant (Escobedo) *Ae. aegypti* strains. The essential oil, dominated by camphor (29.6%), 1,8-cineole (20.8%), and limonene (16.7%), was assessed through laboratory and semi-field bioassays. Larvicidal activity yielded LC_50_ values of 184.38 µg mL^−1^ for the susceptible strain and 305.04 µg mL^−1^ for the resistant strain, with a resistance ratio of 1.65, indicating susceptibility. Oviposition deterrence was quantified using the Oviposition Activity Index (OAI), and median repellent concentrations (RC_50_) were estimated. Under laboratory conditions, RC_50_ values were 1.65 µg mL^−1^ for the NO strain and 1.73 µg mL^−1^ for the Escobedo strain. Under semi-field conditions, the RC_50_ for the Escobedo strain decreased to 0.62 µg mL^−1^. Deterrent activity increased with concentration and persisted for up to 40 days, particularly at higher doses. These results demonstrate that *S. munzii* essential oil exhibits both larvicidal and oviposition deterrent activity against *Ae. aegypti*, including a pyrethroid-resistant population, under laboratory and semi-field conditions. The findings support further evaluation of *S. munzii* essential oil as a potential complementary tool for integrated vector management strategies.

## 1. Introduction

Vector-borne diseases such as dengue, Zika, and chikungunya remain important public health problems in Mexico, where *Aedes aegypti* is widely distributed in urban and peri-urban environments [1,2]. Control programs rely heavily on chemical insecticides; however, the sustained use of larvicides and adulticides has contributed to the widespread emergence of insecticide resistance in *Ae. aegypti* populations across the country [3,4].

Pyrethroid resistance in *Ae. aegypti* is widespread in Mexico and represents one of the main challenges for vector control programs [5,6,7,8,9,10]. Resistance is associated with both target-site mutations and enhanced metabolic detoxification mechanisms [11,12,13,14,15]. In addition, reduced susceptibility to other insecticides used in vector control programs, including temephos and organophosphates, has also been reported in field populations [16,17,18]. These resistance patterns highlight the need for alternative or complementary approaches for mosquito control.

The widespread and multifactorial nature of these resistance patterns highlights the limitations of relying exclusively on insecticide-based control and supports the need to evaluate complementary approaches within integrated vector management strategies. Despite the continued use of larvicides, adulticides, environmental management, and social mobilization, current control strategies have not been consistently effective in preventing arboviral outbreaks [19]. In this context, increasing attention has been directed toward approaches that target mosquito behavior rather than survival.

Oviposition site selection in mosquitoes is mediated by chemical cues associated with water quality, microorganisms, plant-derived compounds, and conspecific immature stages, which can either stimulate or inhibit egg-laying behavior [20]. Volatile organic compounds emitted by aromatic plants can interfere with these sensory processes and modify mosquito behavior through attraction or repellence [21,22]. Because oviposition deterrents act on behavior rather than lethality, they may impose lower selection pressure for resistance and could remain effective in mosquito populations resistant to multiple insecticide classes [23]. Consequently, deterrent-based approaches have gained attention as complementary tools within integrated vector management programs. Laboratory and semi-field assays comparing egg-laying responses between treated and untreated substrates are commonly used to evaluate oviposition deterrence under controlled conditions [23,24].

Botanical products have gained increasing attention in vector control research due to their diverse bioactive constituents and potential activity against insecticide-resistant mosquito populations [25,26]. Among these, essential oils derived from aromatic plants may affect mosquito behavior and survival through larvicidal, repellent, or oviposition deterrent activity depending on their chemical composition [27,28]. Essential oils from several plant species have demonstrated larvicidal and oviposition deterrent activity against *Ae. aegypti* [29,30]. However, relatively few studies have simultaneously evaluated larvicidal and oviposition deterrent effects against resistant populations or incorporated semi-field validation [31].

The genus *Salvia* (Lamiaceae) is one of the most diverse and chemically rich genera of aromatic plants, with Mexico recognized as a major center of diversification and endemism [32]. Several *Salvia* species have been reported to exhibit insecticidal, repellent, or deterrent activity against agricultural and public health pests, effects largely attributed to their essential oils and diterpenoid compounds [25,26,33]. Despite this growing body of evidence, important knowledge gaps remain. Few studies have simultaneously evaluated oviposition deterrent and larvicidal effects within the same experimental framework, and most assessments have been restricted to laboratory conditions or susceptible reference strains. Moreover, information on the performance of *Salvia* essential oils against insecticide-resistant mosquito populations, as well as studies integrating laboratory and semi-field evaluations, remains limited.

The aim of this study was to evaluate the oviposition deterrent and larvicidal effects of *Salvia munzii* essential oil against *Ae. aegypti* using laboratory and semi-field assays. Specifically, we (i) determined larvicidal dose–response parameters under laboratory conditions, (ii) determined dose–response parameters for oviposition deterrence using the Oviposition Activity Index (OAI), (iii) assessed the persistence of the deterrent effect under laboratory conditions, and (iv) evaluated oviposition deterrence under small-scale field (semi-field) conditions. All assays were conducted using the susceptible New Orleans strain and a field strain resistant to pyrethroids (deltamethrin and permethrin) and temephos.

## 2. Materials and Methods

### 2.1. Plant Material, Essential Oil Extraction, and Chemical Characterization of Salvia munzii

Plant material of *S. munzii* was collected from wild populations in Baja California, Mexico, near Ensenada (31°51′55″ N, 116°38′34″ W; 100 m a.s.l.). Sampling was conducted during winter to capture the essential oil profile under low-temperature and reduced photoperiod conditions, which are known to influence terpene biosynthesis in Salvia species [34]. Botanical identification was confirmed by specialists at the Herbarium of the Facultad de Ciencias, Universidad Autonoma de Baja California (Ensenada campus), where a voucher specimen was deposited (BCMEX8717).

Leaves were air-dried in the shade at room temperature and ground before extraction. Essential oil was extracted from 500 g of dried plant material through hydrodistillation for 3 h using a modified Clevenger-type apparatus (Sigma-Aldrich, St. Louis, MO, USA), following European Pharmacopoeia guidelines. The oil was separated, dried over anhydrous sodium sulfate, and stored in amber vials under nitrogen at 4 °C until bioassay analysis.

The essential oil used in this study is from a batch previously characterized under the same conditions by the same research group [35]. Including its chemical profile here offers direct context for understanding the biological activity evaluated in this work. Chemical composition was analyzed using gas chromatography–mass spectrometry (GC–MS) with an Agilent 7890A system (Agilent Technologies, Santa Clara, CA, USA) connected to a 5975C mass selective detector and equipped with an HP-5MS capillary column (Agilent Technologies, Santa Clara, CA, USA) (30 m × 0.25 mm, 0.25 µm film thickness). Helium served as the carrier gas at a flow rate of 1.0 mL min^−1^. The oven temperature program ranged from 70 °C to 250 °C with ramp rates of 2 °C min^−1^ and 10 °C min^−1^. The mass spectrometer operated in electron impact mode (70 eV), scanning from 40 to 400 *m/z*. Compound identification was based on comparing mass spectra to the NIST library and matching calculated Kovats retention indices with literature values, using a homologous series of n-alkanes (C8–C20). Relative abundances were calculated as percentage peak areas without correction factors. Compounds were categorized into monoterpene hydrocarbons, oxygenated monoterpenes, sesquiterpene hydrocarbons, and oxygenated sesquiterpenes.

### 2.2. Aedes aegypti Strains

*Ae. aegypti* from Escobedo, Nuevo Len, northeastern Mexico (25°48′04.0″ N 100°19′25.0″ W), was sampled in 2023 at multiple sites within the municipality. Larvae were collected from at least ten natural breeding containers per site, yielding approximately 1300–1600 individuals at each sampling site. Field material was transported to the Medical Entomology Laboratory, Facultad de Ciencias Biologicas, Universidad Autonoma de Nuevo Leon. A laboratory-maintained susceptible reference strain, New Orleans (NO), reared continuously since 2000, was included.

Larval rearing was carried out using dechlorinated tap water in plastic trays, with nutrition supplied as finely ground bovine liver (MP Biomedicals, LLC, Santa Ana, CA, USA). Once pupation occurred, pupae were individually transferred to 250 mL containers and placed inside 30 cm^3^ mesh cages to allow adult emergence. Emerged adults were provided continuous access to a 10% sucrose solution, and females were subsequently offered an artificial blood meal consisting of lamb blood (*Ovis orientalis*) to induce egg production. Egg collection was facilitated by oviposition cups containing dechlorinated water and lined with filter paper. All colonies were reared under controlled insectary conditions (28 ± 1 °C, 70–80% relative humidity, and a 12:12 h light: dark cycle).

### 2.3. Resistance Characterization of the Field Strain

The resistance status of the Escobedo *Ae. aegypti* field strain was characterized to provide biological context for the deterrent and larvicidal assays. Susceptibility to temephos was evaluated in late third–early fourth instar larvae using the WHO discriminating concentration (DC) (0.012 mg/L) [36]. Pyrethroid resistance was assessed in F_1_ adult females using WHO tube tests with permethrin DC (0.4%) and deltamethrin DC (0.03%) (ChemService, West Chester, PA) [37]. For both insecticide classes, resistance status was classified according to WHO criteria [38]: ≥98% mortality indicates susceptibility; 90–97% suggests possible resistance; <90% confirms resistance.

In parallel, the frequency of the kdr mutations V410L, V1016I, and F1534C in the voltage-gated sodium channel gene was determined in the Escobedo field strain by allele-specific quantitative PCR, following previously validated protocols [10]. Detoxification enzymes were evaluated through biochemical assays measuring the activity of α- and β-esterases, cytochrome P450 monooxygenases, and glutathione S-transferases in both larvae and adult females from the Escobedo field strain and the susceptible NO reference strain, using standardized colorimetric methods [39,40,41,42,43]. Results of these analyses are presented to document the resistant phenotype of the Escobedo strain.

### 2.4. Laboratory Evaluation of Larvicidal Activity

Larvicidal bioassays were performed according to the WHO standard protocol [44]. Assays were conducted in 250 mL plastic cups containing test solutions prepared with Tween-80 (0.001%) (Merck, S.A de C.V, Estado de Mexico. Mexico) as an emulsifier. For the NO strain, eleven concentrations ranging from 70 to 500 µg mL^−1^ were evaluated, whereas for the Escobedo strain, ten concentrations ranging from 100 to 650 µg mL^−1^ were tested. A control consisting of dechlorinated water containing Tween-80 (0.001%) was included in each experiment. For each concentration and the control, three replicates were performed using 20 late third–early fourth instar larvae per replicate for both strains. Larval mortality was recorded 24 h post-exposure, and larvae were considered dead if they showed no movement after gentle probing. When control mortality was 5–20%, results were corrected; assays with control mortality > 20% were discarded [45]. Mortality data were subjected to probit analysis to estimate LC_50_ and LC_90_ values and their corresponding confidence intervals. Resistance ratio (RR) was calculated by dividing the LC_50_ of the field population by that of the susceptible New Orleans strain. The level of resistance was interpreted according to the criteria of Mazzarri and Georghiou [46]: RR < 5 indicates susceptibility, RR = 5–10 indicates moderate resistance, and RR > 10 indicates high resistance.

### 2.5. Laboratory Evaluation of Oviposition Deterrence

Laboratory oviposition deterrence bioassays were conducted following the methodology described by Xue et al. [47] to determine the effective concentrations of *S. munzii* essential oil against gravid *Ae. aegypti* females. The essential oil was emulsified in distilled water using Tween-80 (0.001%) as a surfactant and evaluated over a concentration range of 0.1–15 ppm.

Bioassays were performed using black plastic ovitraps (200 mL capacity). Each experimental unit consisted of two ovitraps: one containing the test solution and one containing the control solution (water + Tween-80), each with a final volume of 100 mL. Ovitraps were placed diagonally in opposite corners of a BioQuip^®^ mesh cage (30 × 30 × 30 cm) (BioQuip Products, Rancho Dominguez, CA, USA), and their positions were alternated between replicates to minimize positional effects.

A strip of filter paper (24 × 8 cm) was placed inside each ovitrap as an oviposition substrate, ensuring continuous contact with the liquid to maintain moisture. Fifteen gravid *Ae. aegypti* females (10 days old, 5 days post-blood feeding) were released into each cage and provided with a 10% sucrose solution. After 24 h of exposure, the number of eggs deposited on each substrate was recorded.

Each concentration was tested in four independent replicates for both the Escobedo strain and the susceptible NO strain.

The behavioral response of gravid females was quantified using the Oviposition Activity Index (OAI) [48]:

$$OAI=\frac{N_{T}-N_{C}}{N_{T}+N_{C}}$$

where N_T_ represents the number of eggs laid in the treated ovitrap and N_C_ the number of eggs laid in the control. OAI values range from −1 to +1, with negative values indicating deterrence, positive values attraction, and values near zero indicating neutrality. Additionally, the effective repellency percentage (ER) was calculated for each concentration using the formula:

$$\%ER=\frac{N_{C}-N_{T}}{N_{C}}\times 100$$

where N_C_ is the number of eggs laid in the control ovitrap, and N_T_ is the number of eggs laid in the treated ovitrap. The median repellent concentration (RC_50_) and its 95% confidence interval were estimated from ER (%) values using Probit analysis in PoloPlus^®^ v2.0 (LeOra Software, Berkeley, CA, USA).

### 2.6. Laboratory Evaluation of Oviposition Deterrence Persistence

A second series of laboratory bioassays was conducted to assess the persistence of the oviposition deterrent activity of *S. munzii* essential oil. Persistence was evaluated at the RC_50_ concentration as well as at the concentrations that produced the highest oviposition deterrence (10 and 15 µg mL^−1^) in both strains.

Experimental conditions and procedures were identical to those described for the initial oviposition deterrence assays. For persistence evaluation, a new cohort of 15 gravid females was introduced into each cage every 5–6 days and allowed to oviposit for 24 h before removal. After each exposure period, test containers were refilled to 100 mL with the corresponding solution and fitted with a new oviposition substrate.

Successive bioassays were conducted using the same containers for a total duration of 40 days, for both the Escobedo and the NO strain. The 40-day endpoint was selected to evaluate persistence over a timeframe considered operationally relevant for potential vector control applications. This protocol represents a modification of the method described by Xue et al. [47].

OAI values were calculated for each concentration, strain, and exposure period to evaluate temporal changes in deterrent efficacy.

### 2.7. Semi-Field Evaluation of Oviposition Deterrence

Semi-field oviposition deterrence assays were conducted in an outdoor mesh cage (3 m × 3 m × 2.77 m) following the protocol described by Xue et al. [47]. Black ovitraps (500 mL capacity) containing 200 mL of test solution and a filter paper strip as oviposition substrate were used.

Treatments consisted of four concentrations of *S. munzii* essential oil (0.5, 2, 10, and 40 ppm) and an untreated control containing water only. Bioassays were conducted exclusively with the Escobedo field population.

Ovitraps were randomly positioned inside the cage, and their positions were alternated between replicates to minimize positional effects. One hundred gravid females were released weekly. Traps remained in place for 48 h, after which filter papers were collected and eggs counted. The experiment was replicated four times.

Data analysis included estimation of median repellent concentration (RC_50_) and calculation of OAI values.

## 3. Results

### 3.1. Chemical Composition of S. munzii Essential Oil

The chemical composition of the *S. munzii* essential oil used in this study is shown in Table 1 and matches the profile previously reported for winter-collected material [35]. This composition is provided to support the interpretation of the biological activity evaluated in the present work.

The essential oil was characterized by a predominance of monoterpenes, especially oxygenated monoterpenes, which are generally linked to biological activity in aromatic plants. Camphor (29.6%) and 1,8-cineole (20.8%) were identified as the main components, followed by limonene (16.7%) and caryophyllene (9.8%). This monoterpene-dominated profile is commonly associated with essential oils exhibiting biological activity against insects. The GC–MS chromatogram of the *S. munzii* essential oil is provided as Supplementary Figure S1.

### 3.2. Resistance in Ae. aegypti Escobedo Strain

Susceptibility assays conducted at DCs revealed low mortality levels in the Escobedo strain of *Ae. aegypti* for both larval and adult stages (Table 2). Larval exposure to the organophosphate temephos resulted in 17% mortality, indicating resistance to this larvicide. Adult bioassays showed similarly reduced susceptibility to pyrethroid insecticides, with mortality values of 45% for permethrin and 47% for deltamethrin. According to established diagnostic criteria, the Escobedo population was classified as resistant to temephos, permethrin, and deltamethrin.

**Figure 1:**
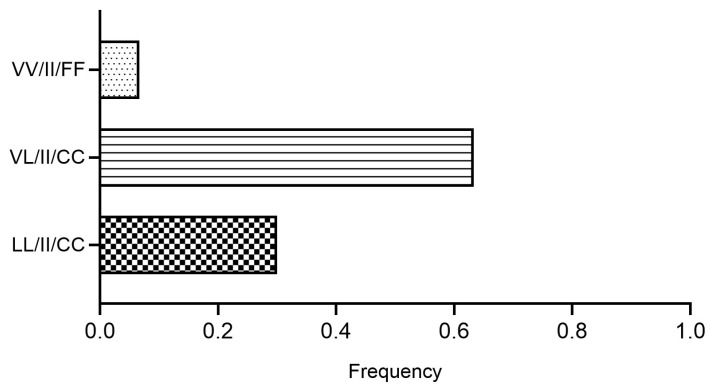
Frequency of combined kdr genotypes (V410L, V1016I, and F1534C) in the Escobedo strain of *Aedes aegypti*.

The distribution of combined kdr genotypes associated with pyrethroid resistance was determined in the Escobedo strain (Figure 1). The genotype VL/II/CC was the most frequent (0.63), followed by the triple resistant LL/II/CC (0.30), whereas VV/II/FF was present at a low frequency (0.07).

The activity of detoxification enzymes was evaluated in larvae from the Escobedo strain and compared with the susceptible NO strain (Table 3). The activity of α-esterases was significantly higher in the Escobedo strain than in the NO strain (*p* ≤ 0.001). The β-esterase activity also showed a significant increase in Escobedo larvae (*p* ≤ 0.05). In contrast, cytochrome P450 monooxygenase activity did not differ significantly between strains. Glutathione S-transferase (GST) activity was significantly higher in the Escobedo strain compared with the susceptible strain (*p* ≤ 0.001).

In adult females, detoxification enzyme activity was significantly higher in the Escobedo strain than in the susceptible NO strain (Table 2), with α-esterases, β-esterases, cytochrome P450 monooxygenases, and glutathione S-transferases (GST) all significantly elevated (*p* ≤ 0.01).

### 3.3. Larvicidal Activity of S. munzii Essential Oil

The essential oil of *S. munzii* exhibited larvicidal activity against both *Ae. aegypti* strains (Table 4). Probit analysis estimated an LC_50_ of 184.38 µg mL^−1^ (95% CI: 171.43–198.15) for the New Orleans strain and 305.04 µg mL^−1^ (95% CI: 289.23–321.17) for the Escobedo population. The LC_90_ values were 282.28 µg mL^−1^ (95% CI: 254.88–328.02) and 516.06 µg mL^−1^ (95% CI: 479.14–565.39) for New Orleans and Escobedo, respectively.

The non-overlapping confidence intervals for LC_50_ and LC_90_ indicate that the NO strain was significantly more susceptible to the essential oil than the Escobedo strain. However, the RR_50_ of 1.65 indicates that the Escobedo strain is susceptible according to established criteria [46].

### 3.4. Oviposition Deterrence Under Laboratory Conditions

The oviposition deterrent activity of *S. munzii* essential oil increased with concentration in both *Ae. aegypti* strains (Table 5; Tables S1 and S2). In the NO strain, OAI values decreased progressively from −0.005 at 0.1 µg mL^−1^ to −1.000 at 15 µg mL^−1^, indicating a strong deterrent effect at higher concentrations. A similar pattern was observed in the Escobedo strain, where OAI values ranged from −0.004 at 0.1 µg mL^−1^ to −1.000 at 15 µg mL^−1^.

Probit analysis estimated a median repellent concentration (RC_50_) of 1.65 µg mL^−1^ (95% CI: 1.13–2.41) for the NO strain and 1.73 µg mL^−1^ (95% CI: 1.02–2.88) for the Escobedo strain. The overlap between the confidence intervals indicates that the RC_50_ values did not differ significantly between strains. The slopes of the Probit models were 1.48 ± 0.083 for New Orleans and 1.56 ± 0.088 for Escobedo, with χ^2^ values of 38.48 (df = 8) and 36.42 (df = 6), respectively.

### 3.5. Oviposition Deterrence Persistence Under Laboratory Conditions

**Figure 2:**
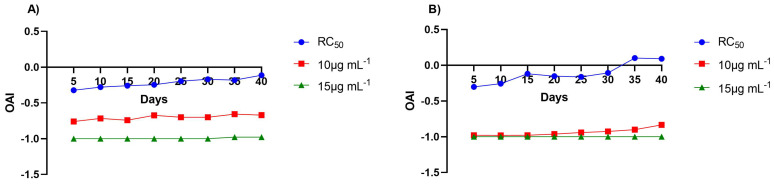
Persistence of oviposition deterrence of *Salvia munzii* essential oil against *Aedes aegypti* under laboratory conditions expressed as oviposition activity index (OAI) values over a 40-day period. Evaluations were performed at the RC_50_ and at two higher concentrations (10 and 15 µg mL^−1^) in the New Orleans (**A**) and Escobedo (**B**) strains.

The persistence of oviposition deterrence of *S. munzii* essential oil was evaluated for up to 40 days in both *Ae. aegypti* strains (Figure 2; Table S3). In the NO strain, the deterrent effect at the RC_50_ gradually declined over time, although OAI values remained negative throughout the experiment, indicating that deterrence persisted 40 days. In contrast, higher concentrations maintained stronger deterrent effects, with 10 µg mL^−1^ showing consistent deterrence and 15 µg mL^−1^ producing nearly complete oviposition deterrence during the entire evaluation period.

In the Escobedo strain, deterrence at the RC_50_ decreased more rapidly, and OAI values became positive after 35–40 days, indicating loss of deterrent activity at this concentration. However, the higher concentrations maintained strong oviposition deterrence, particularly at 15 µg mL^−1^, where OAI values remained close to −1 throughout the 40-day period.

### 3.6. Oviposition Deterrence Under Semi-Field Conditions

Under semi-field conditions, *S. munzii* essential oil showed oviposition deterrent activity against the *Ae. aegypti* Escobedo strain (Table 6; Tables S4 and S5). OAI values decreased with increasing concentration, ranging from −0.313 at 0.5 µg mL^−1^ to −1.000 at 40 µg mL^−1^, indicating a strong deterrent effect at higher concentrations.

Probit analysis estimated a median repellent concentration (RC_50_) of 0.62 µg mL^−1^ (95% CI: 0.31–0.98). The lowest concentration produced moderate deterrence, whereas higher concentrations (10 and 40 µg mL^−1^) resulted in strong oviposition deterrence, with OAI values close to −1. The RC_50_ estimated under semi-field conditions was lower than that obtained in laboratory assays for the same population (1.73 µg mL^−1^), indicating higher deterrent efficacy under semi-field conditions.

## 4. Discussion

### 4.1. Insecticide Resistance in the Ae. aegypti Escobedo Strain

The Escobedo strain showed resistance to both larvicides and adulticides, with mortality values of 17% for temephos, 45% for permethrin, and 47% for deltamethrin, confirming a multi-insecticide resistance phenotype. Similar resistance patterns have been widely reported in *Ae. aegypti* s from Mexico, where reduced susceptibility to pyrethroids has been associated with sustained insecticide pressure in vector control programs [6,10,49].

The predominance of the combined kdr genotype VL/II/CC observed in this study is consistent with previous reports describing the widespread co-occurrence of the V410L, V1016I, and F1534C mutations in Mexican populations of *Ae. aegypti*. These multilocus genotypes have been associated with increased resistance levels and reduced knockdown sensitivity to pyrethroids [10,50].

Biochemical assays revealed increased activity of detoxification enzymes, including esterases and GST in larvae, and elevated activity of all enzymes evaluated in adults. The coexistence of metabolic detoxification mechanisms and target-site mutations has been frequently reported in resistant *Ae. aegypti* populations. Nationwide analyses in Mexico have demonstrated that pyrethroid resistance is associated with the combined presence of kdr mutations and increased activity of detoxification enzymes, resulting in complex resistance profiles [10]. Similar patterns involving multiple resistance mechanisms, including enhanced esterase, GST, and monooxygenase activity together with sodium channel mutations, have also been documented in *Ae. aegypti* populations from other regions. These combined mechanisms may contribute to higher resistance levels than those conferred by single mechanisms [51,52].

Interestingly, cytochrome P450 monooxygenase activity was significantly elevated only in adult females of the Escobedo strain, whereas no significant increase was detected in larvae. This stage-specific pattern may reflect differences in physiological requirements and detoxification demands between immature and adult stages. Adult females are more frequently exposed to pyrethroid-based vector control interventions, which may favor the selection or induction of detoxification pathways associated with P450 activity. Previous studies have shown that the expression of cytochrome P450 genes can vary across developmental stages in mosquitoes, with some resistance-associated P450 genes differentially expressed between larvae and adults [53].

Resistance to temephos observed in the Escobedo population is consistent with previous studies documenting reduced susceptibility to this larvicide in Mexico, where resistance has been associated with prolonged use in larval control programs [18].

### 4.2. Larvicidal Activity of S. munzii Essential Oil

The present study demonstrated that *S. munzii* essential oil exhibited moderate larvicidal activity against *Ae. aegypti*, with LC_50_ values of 184.38 µg/mL for the susceptible NO strain and 305.04 µg/mL for the insecticide-resistant Escobedo strain after 24 h exposure. These results position *S. munzii* within the intermediate range of efficacy reported for Lamiaceae essential oils, a family extensively studied for its insecticidal properties against mosquito vectors.

The Lamiaceae family exhibits remarkable diversity in larvicidal potency. Among the most potent species, *Plectranthus mollis* demonstrated exceptional activity with an LC_50_ of 25.39 µg/mL [54], while *Mentha × villosa* showed an LC_50_ of 33.9 µg/mL [55], and *Mentha pulegium* exhibited an LC_50_ of 38.75 µg/mL [56]. Other species demonstrated intermediate activity, including *Mentha piperita* (LC_50_ = 47.58–165.57 µg/mL), depending on chemotype [56,57], *Mentha spicata* (LC_50_ = 56.08 µg/mL) [58], and various *Ocimum* species ranging from 60 to 148.5 µg/mL [56,59,60]. In comparison, *S. munzii* exhibited lower potency than these highly active species but remained substantially more effective than weak performers such as *Vitex ovata* (LC_50_ = 2114 µg/mL) [61] as well as *Leucas aspera* and *Hyptis suaveolens* extracts (LC_50_ = 483–1443 µg/mL) [62,63].

The larvicidal activity of *S. munzii* can be attributed to its monoterpene-rich composition, dominated by camphor (29.6%), 1,8-cineole (20.8%), and limonene (16.7%), with additional contributions from α-pinene (8.2%), β-pinene (5.2%), and camphene (3.9%). This chemical profile combines three of the most extensively studied larvicidal monoterpenes in the Lamiaceae literature. Camphor, a bicyclic monoterpenoid ketone, has been associated with strong larvicidal activity in several species. Other Lamiaceae oils containing camphor-related ketones have demonstrated notable efficacy, including *Mentha longifolia* with piperitone oxide (LC_50_ = 46.7 µg/mL) [64] and *M. spicata* with carvone (LC_50_ = 56.08 µg/mL) [58]. Ketone-containing monoterpenes have been suggested to contribute to high larvicidal activity through their lower volatility and synergistic effects with other components [64].

The presence of 1,8-cineole (20.8%) as the second major component is particularly significant, as this compound has been identified as a key larvicidal constituent in several highly active Lamiaceae species. *Ocimum gratissimum*, which contains 1,8-cineole as a major component, exhibited an LC_50_ of 60 µg/mL against *Ae. aegypti* [59]. *Lavandula stoechas*, containing 25.16% 1,8-cineole, demonstrated an LC_50_ of 112.51 µg/mL against *Anopheles* larvae [65]. The combination of camphor and 1,8-cineole in *S. munzii* provides a dual mechanism of action involving both neurotoxic (camphor) and membrane-disrupting (1,8-cineole) effects.

Limonene (16.7%), the third major component of *S. munzii*, is one of the most prevalent and active monoterpenes in Lamiaceae essential oils. Species rich in limonene have demonstrated strong larvicidal activity. For example, *M. × villosa* containing 8.75% limonene showed larvicidal activity against *Ae. agypti* (LC_50_ = 33.9 µg/mL) [55], while *M. spicata* with 11.30% limonene exhibited similar effects (LC_50_ = 56.08 µg/mL) [58]. Likewise, *Lavandula gibsoni* essential oil presented larvicidal activity against *Ae. aegypti* (LC_50_ = 48.32 µg/mL) [54], and *M. piperita* showed activity against *Culex pipiens* (LC_50_ = 47.58 µg/mL [56]. The presence of limonene at 16.7% in *S. munzii* represents a higher concentration than in many of these more potent species, suggesting that the moderate activity of *S. munzii* is not due to the absence of active monoterpenes but rather to the specific ratio and synergistic interactions among components.

The monoterpene hydrocarbons α-pinene (8.2%), β-pinene (5.2%), and camphene (3.9%) provide additional larvicidal activity through membrane disruption mechanisms. *Ocimum basilicum* containing L-β-pinene exhibited an LC_50_ of 73.45 µg/mL against *Cx. pipiens* [56], while various *M. piperita* chemotypes containing pinenes showed variable activity [66]. These monoterpene hydrocarbons are thought to act primarily through physical mechanisms, dissolving in lipid bilayers and causing structural disorganization and increased membrane permeability [67].

Essential oils function as complex mixtures where synergistic interactions among components determine overall efficacy. Lima et al. [55] demonstrated that complete *M. × villosa* oil exhibited significantly greater activity than its isolated major component, confirming that minor constituents and synergistic effects contribute substantially to toxicity. Similarly, El-Kasem Bosly [67] proposed that synergistic effects of compounds within essential oils enhance their biological activity. The moderate activity of *S. munzii* despite containing three highly active monoterpenes (camphor, 1,8-cineole, limonene) at substantial concentrations suggests that the specific ratio of these components may result in suboptimal synergy compared to more potent species. The variable activity observed among different *M. piperita* chemotypes (LC_50_ ranging from 38.75 to 414.6 µg/mL) further illustrates that compositional balance is critical [56,66]. Future fractionation studies examining individual components and defined binary or ternary mixtures could elucidate the specific interactions among camphor, 1,8-cineole, and limonene in *S. munzii*.

A critical finding of this study is that *S. munzii* retained significant activity against the insecticide-resistant Escobedo strain, with a RR_50_ of 1.65. This is particularly relevant because previous studies evaluating Lamiaceae essential oils have generally been conducted using laboratory-susceptible strains or field populations with unknown resistance status. As a result, information on the efficacy of these botanical products against insecticide-resistant mosquito populations remains limited. The reduced cross-resistance observed with *S. munzii* is consistent with the multi-target mode of action characteristic of essential oils, which has been proposed to reduce the chances of resistance development compared to synthetic insecticides acting on single molecular targets [64,67,^68^]. Essential oils exert toxicity through multiple mechanisms, and this complexity limits the effectiveness of resistance mechanisms such as enhanced detoxification or target-site mutations. While the Escobedo population exhibits elevated detoxification enzyme activity and multiple kdr mutations conferring pyrethroid resistance, these mechanisms would be expected to provide only partial protection against the structurally diverse monoterpenes in *S. munzii* oil. Norris et al. [68] demonstrated that plant terpenoids act at a variety of molecular targets, many of which are distinct from those of conventional insecticides, suggesting little chance of cross-resistance development with compounds currently available on the market. Furthermore, Norris et al. [69] showed that multiple plant essential oils significantly inhibited both cytochrome P450-dependent monooxygenase and glutathione S-transferase activities in *Ae. aegypti*, indicating that essential oils can overcome metabolic resistance mechanisms by inhibiting the very detoxification enzymes upregulated in resistant populations.

The resistance ratio of 1.65 observed for larvicidal activity indicates that the Escobedo strain, despite documented pyrethroid resistance, remains susceptible to *S. munzii* essential oil. However, the slightly higher LC_50_ in the resistant strain warrants further investigation. Pyrethroid-resistant *Ae. aegypti* populations frequently exhibit elevated activity of detoxification enzymes, including cytochrome P450 monooxygenases, carboxylesterases, and glutathione S-transferases (GSTs), which may influence responses to structurally diverse xenobiotics [70,71]. Cytochrome P450 enzymes, in particular, play an important role in the metabolism of insecticides and other foreign compounds in mosquitoes [72]. However, formal cross-resistance testing or synergist bioassays were not conducted in the present study, and therefore the possibility of metabolic interactions remains speculative and requires further investigation.

The larvicidal mechanisms of *S. munzii* likely involve multiple complementary pathways. The insecticidal activities of essential oils are attributed to their monoterpene and sesquiterpene constituents, acting through diverse mechanisms. Camphor and other oxygenated monoterpenes interfere with insect nervous system function. The monoterpene hydrocarbons including limonene, α-pinene, β-pinene, and camphene disrupt lipid membranes, causing cellular dysfunction and osmotic imbalance [67]. The compound 1,8-cineole is known for its ability to penetrate biological membranes and interfere with respiratory function. Essential oils may also form surface films that impede larval respiration and inhibit mitochondrial function. Additionally, monoterpenes can induce oxidative stress and contribute to larval mortality through multiple complementary pathways. This multi-target nature of essential oils has been further supported [68,69], demonstrating that different plant terpenoids exhibit varying levels of propensity for interfering with specific enzyme systems, and that synergistic profiles of plant essential oils are highly specific to the target insect strain and the particular combination of compounds present. The observation that multiple mechanisms of action operate simultaneously suggests that resistance development to essential oils would require concurrent mutations affecting multiple physiological systems, a scenario far less likely than resistance to single-target synthetic insecticides [69].

The moderate larvicidal activity of *S. munzii*, combined with its efficacy against resistant strains, suggests potential applications in integrated vector management. Essential oils offer advantages including lower environmental persistence, reduced non-target impacts, and reduced resistance risk due to their complex composition [64,67,68]. However, the moderate LC_50_ values (184.38–305.04 µg/mL) are higher than conventional larvicides, necessitating formulation strategies such as microencapsulation to enhance stability and efficacy. Martins et al. [73] demonstrated that microencapsulation of *Melissa officinalis* essential oil reduced the LC_50_ from 40.6 to 22.1 µg/mL, representing a 1.8-fold improvement in potency. Similar formulation approaches could potentially enhance the efficacy of *S. munzii* oil. Field trials are essential to assess operational feasibility, cost-effectiveness, and environmental impact under realistic conditions.

### 4.3. Oviposition Deterrent Activity of Salvia munzii Essential Oil

The present study demonstrated that *S. munzii* essential oil exhibited strong oviposition deterrent activity against *Ae. aegypti*, with RC_50_ values of 1.65 µg/mL for the susceptible NO strain and 1.73 µg mL^−1^ for the insecticide-resistant Escobedo strain under laboratory conditions. Notably, under semi-field conditions, the RC_50_ for the Escobedo strain decreased to 0.62 µg mL^−1^, indicating enhanced deterrent efficacy in a more realistic environmental setting. At the highest tested concentration (40 µg mL^−1^), complete oviposition deterrence (OAI = −1.0) was achieved under semi-field conditions, demonstrating the potential of *S. munzii* essential oil as a highly effective oviposition deterrent.

The evaluation of plant essential oils as alternatives or complements to synthetic insecticides has generated a substantial body of literature, yet relatively few botanical products have achieved operational deployment in vector control programs [74,75]. The present study contributes to this field by integrating chemical characterization, dual-strain testing (susceptible and pyrethroid-resistant), semi-field validation, and persistence assessment within the same experimental framework. The inclusion of a pyrethroid-resistant field strain provides information relevant to cross-resistance considerations in *Ae. aegypti* populations [76], while the semi-field oviposition assays and the 40-day persistence assessment provide ecologically relevant information that remains limited in the botanical insecticide literature [77,78].

The concentration-dependent oviposition deterrent response observed for *S. munzii* is consistent with previous studies on essential oils against *Ae. aegypti* [79]. The deterrent activity of essential oils from *M. piperita*, *O. basilicum*, *Rosmarinus officinalis* (Lamiaceae), *Cymbopogon nardus* (Poaceae), and *Apium graveolens* (Apiaceae) decreased as concentration declined, with highly negative OAI values and elevated effective repellency at higher doses. For example, *M. piperita*, *O. basilicum*, and *C. nardus* showed strong deterrent effects at 10% concentration, whereas activity decreased at lower concentrations. This pattern agrees with the present results, in which oviposition deterrence by *S. munzii* intensified progressively with increasing concentration under both laboratory and semi-field conditions.

Within the Lamiaceae family, essential oils of *M. spicata* and *O. basilicum* showed oviposition deterrence only at relatively high concentrations [80]. Similarly, *O. basilicum* evaluated at 1%, 5%, and 10% produced OAI values that increased with concentration but did not reach complete inhibition [81]. Strong deterrence was also reported for *Salvia officinalis* (OAI = −0.94 at 200 mg/L), although complete inhibition was not achieved [82]. In contrast, *S. munzii* reached complete oviposition deterrence (OAI = −1.0) at 40 µg mL^−1^ under semi-field conditions and at substantially lower concentrations, suggesting greater potency within this botanical group.

Beyond the Lamiaceae family, similar comparisons further highlight the strong activity of *S. munzii*. Essential oils from *Alpinia galanga* (Zingiberaceae), *Anethum graveolens*, *Foeniculum vulgare*, and *Pimpinella anisum* (Apiaceae) showed concentration-dependent deterrence, with only *A. graveolens* achieving complete inhibition (OAI = −1.0) at 10% concentration, whereas the remaining oils reached maximum OAI values between −0.8 and −0.9 [83]. Strong deterrence was also reported for *Lippia alba* and *Lippia origanoides* (Verbenaceae), with OAI values ranging from −0.83 to −0.93, and for *Eucalyptus citriodora* (Myrtaceae), which reached complete inhibition (OAI = −1.0) only at 200 ppm [84]. Oviposition reduction for *Etlingera elatior* (Zingiberaceae) at 100 ppm and deterrence values ranging from 16.6% to 94.7% across multiple plant species have also been reported [85,86]. These studies indicate that complete oviposition deterrence is uncommon and generally requires high concentrations. In contrast, *S. munzii* achieved complete oviposition deterrence at 40 µg mL^−1^ under semi-field conditions, indicating comparable or higher efficacy at lower concentrations.

Consistent with this interpretation, significant reductions in egg deposition have been reported for several essential oils, although none produced complete inhibition under the tested conditions [87]. These findings indicate that, while oviposition deterrence is common among plant-derived oils, complete inhibition is rarely achieved and typically requires high concentrations. In this context, the strong negative OAI values observed for *S. munzii*, exceeding the repellency threshold proposed by de Aguiar et al. [88] (OAI < −0.30), indicate a markedly stronger behavioral response than that reported for most botanical products.

The strong oviposition deterrent activity observed for *S. munzii* may also be related to its monoterpene-rich composition. Previous studies have shown that essential oils containing compounds such as limonene, β-pinene, and 1,8-cineole can influence oviposition behavior in mosquitoes. These monoterpenes contribute to oviposition deterrence and interfere with host-site selection by gravid females [89]. These compounds are present in *S. munzii* essential oil, suggesting that their combined effects contribute to the strong deterrent activity.

A particularly noteworthy finding of the present study is the absence of significant differences in oviposition deterrent activity between the insecticide-susceptible NO strain (RC_50_ = 1.65 µg mL^−1^) and the insecticide-resistant Escobedo strain (RC_50_ = 1.73 µg mL^−1^) under laboratory conditions. This near-equivalent activity suggests that the behavioral mechanisms of oviposition deterrence by *S. munzii* essential oil are independent of the physiological resistance mechanisms that confer tolerance to conventional insecticides. The Escobedo strain exhibits multiple resistance mechanisms, including elevated α-esterase, β-esterase, glutathione S-transferase, and cytochrome P450 monooxygenase activity, as well as high frequencies of kdr mutations (V410L, V1016I, F1534C). The fact that these resistance mechanisms do not compromise the deterrent activity of *S. munzii* essential oil represents a significant practical advantage, as oviposition deterrents can remain effective even in populations with extensive insecticide resistance.

It should also be considered that long-term laboratory colonization may alter behavioral traits relative to field populations through adaptation to laboratory conditions. However, the similar RC_50_ values observed between the NO and Escobedo strains under laboratory conditions suggest that these potential colonization effects did not substantially influence the oviposition deterrent responses observed in the present study.

The enhanced oviposition deterrent activity observed under semi-field conditions (RC_50_ = 0.62 µg mL^−1^) compared to laboratory conditions (RC_50_ = 1.73 µg mL^−1^ for the same Escobedo strain) is particularly significant for practical applications. This approximately 2.8-fold increase in potency under more realistic environmental conditions suggests that factors present in semi-field settings, such as natural airflow patterns, environmental odors, and behavioral complexity, may enhance the deterrent effects of *S. munzii* essential oil. Oviposition deterrence has also been reported to vary among mosquito species and environmental contexts, with *Culex quinquefasciatus* showing greater deterrence than *Ae. aegypti* or *Anopheles gambiae* under comparable conditions [80]. The improved performance of *S. munzii* under semi-field conditions suggests that laboratory bioassays may underestimate the practical efficacy of oviposition deterrents and that field validation is essential for accurate assessment of botanical insecticides.

Essential oils function as complex mixtures in which overall efficacy depends on their chemical composition. Differences in oviposition deterrent activity have been reported among essential oils of *M. spicata*, *O. basilicum*, and *Abutilon indicum*, indicating that bioactivity varies among oils with distinct chemical profiles [80]. Although specific contributions of individual compounds were not isolated, the observed variation in deterrent responses suggests that the combined composition of essential oils influences their effectiveness. In this context, the strong deterrent activity of *S. munzii*, despite sharing major components such as limonene, camphor, 1,8-cineole, and pinenes with other less active oils, may be related to differences in the relative abundance and interaction of these constituents.

From an ecological perspective, the oviposition deterrent activity of *S. munzii* may be related to the natural biological functions of essential oils in *Salvia* species. Essential oils in *Salvia* have been reported to exhibit diverse biological activities, which are associated with their complex chemical composition and interactions among constituents [34]. These plant secondary metabolites are widely recognized as part of plant defense strategies against environmental and biotic stresses. In this context, the strong deterrent effect observed for *S. munzii* may reflect the biological role of these compounds in influencing insect behavior.

Despite the promising laboratory and semi-field activity reported for many botanical products, relatively few essential oil-based mosquito control tools have achieved operational implementation [90,91]. Important limitations include variability in chemical composition associated with geographic origin, plant material, seasonality, and extraction conditions, which complicate standardization and quality control of botanical products [92,93]. In addition, formulation instability and volatility may reduce residual persistence under field conditions unless controlled-release formulations are developed [94,95]. Regulatory approval of botanical insecticides also requires toxicological, environmental, and efficacy evaluations comparable to those required for synthetic insecticides, representing important economic and logistical barriers for product development [90,92]. Although the present study provides chemical characterization, activity data against resistant strains, persistence assessment, and semi-field validation, additional studies addressing formulation development, operational feasibility, and large-scale field performance are still necessary before practical implementation can be considered [77]. Positioning *S. munzii* essential oil within integrated vector management strategies, rather than as a standalone replacement for synthetic insecticides, may represent a more realistic pathway for operational use [74,96].

The practical implications of the present findings are relevant for integrated vector management. Oviposition deterrents target mosquito behavior and may complement conventional insecticides, particularly in areas with insecticide resistance. The high potency of *S. munzii* essential oil, with RC_50_ values in the low µg mL^−1^ range and complete deterrence achieved at moderate concentrations, highlights its potential as a botanical oviposition deterrent.

Several practical applications can be envisioned, including the use of treated oviposition traps or application to domestic water-holding containers to reduce mosquito breeding. The persistence of deterrent activity for up to 40 days suggests that periodic applications could provide sustained protection under field conditions. Additional studies extending beyond 40 days would help determine the maximum duration of deterrent activity and optimize potential reapplication intervals under operational conditions.

However, the implementation of plant-derived deterrents requires careful consideration of variability in essential oil composition. Seasonal and environmental factors can influence the relative abundance of key metabolites such as camphor, 1,8-cineole, and limonene, which may affect biological activity [35]. Therefore, standardization of essential oil composition and development of stable formulations are essential steps prior to operational use. Additional field evaluations are also required to determine effectiveness under real-world conditions.

The dual-choice oviposition assay used in the present study is a standard approach for evaluating relative oviposition preference between treated and untreated substrates [48,97]. This design also allows estimation of oviposition deterrence metrics such as OAI and concentration–response relationships [98]. However, this design primarily measures avoidance behavior and does not distinguish whether reduced egg deposition in treated containers reflects redistribution of oviposition to untreated sites or suppression of total egg output through skip oviposition behavior [99,100]. Skip oviposition may have important ecological implications for mosquito population dynamics and control strategies [101]. Because total egg production and no-choice assays were not evaluated, the present study cannot definitively differentiate between these behavioral responses. Future studies incorporating no-choice assays or both-treated experimental designs would help clarify whether *S. munzii* essential oil suppresses total egg output in addition to deterring oviposition at treated sites [90,93].

## 5. Conclusions

The present study showed that *S. munzii* essential oil exhibited both oviposition deterrent and larvicidal activity against *Ae. aegypti*. The essential oil produced concentration-dependent deterrence in both susceptible and insecticide-resistant strains, with similar RC_50_ values under laboratory conditions and lower RC_50_ values under semi-field conditions. Deterrent activity persisted for up to 40 days, particularly at higher concentrations, indicating prolonged behavioral activity over the evaluation period.

Larvicidal bioassays showed activity against larvae of both strains. Although the resistant Escobedo population exhibited higher LC_50_ values than the susceptible strain, the resistance ratio (RR_50_ = 1.65) indicated susceptibility. However, formal cross-resistance assays were not conducted, and the possibility of metabolic interactions associated with insecticide resistance requires further investigation.

Overall, the combined oviposition deterrent and larvicidal activity observed under laboratory and semi-field conditions supports further evaluation of *S. munzii* essential oil as a potential complementary tool within integrated vector management strategies. Additional studies addressing formulation development, long-term field performance, toxicological evaluation, and operational feasibility are necessary before practical implementation can be considered.

## Figures and Tables

**Table 1:** Chemical composition of the *Salvia munzii* essential oil used in the present study.

Compounds	Calculated RI	Literature RI	Relative Abundance (%)	Formula	Chemical Class
α-Pinene	944	932	8.2	C_10_H_16_	MH
Camphene	959	946	3.9	C_10_H_16_	MH
β-Pinene	990	974	5.2	C_10_H_16_	MH
β-Myrcene	1002	988	0.9	C_10_H_16_	MH
Limonene	1037	1024	16.7	C_10_H_16_	MH
1,8-Cineole	1040	1026	20.8	C_10_H_18_O	MO
Camphor	1143	1141	29.6	C_10_H_16_O	MO
Caryophyllene	1437	1416	9.8	C_15_H_24_	SH
δ-Cadinene	1545	1513	1.2	C_15_H_24_	SH
Borneol	1173	1165	1.9	C_10_H_18_O	MO

Chemical composition previously reported [35]. Percentages correspond to relative peak-area normalization obtained from GC–MS analysis. Chemical classes: MH, monoterpene hydrocarbon; MO, oxygenated monoterpene; SH, sesquiterpene hydrocarbon. RI: retention index (Kovats index).

**Table 2:** Susceptibility of *Aedes aegypti* larvae and females from the Escobedo strain to the discriminating concentrations of the larvicide temephos and the adulticides permethrin and deltamethrin.

Insecticide	Mortality (%)	Status
Temephos	17	Resistant
Permethrin	45	Resistant
Deltamethrin	47	Resistant

**Table 3:** Quantification of detoxification enzyme activity in larvae and adult females of *Aedes aegypti* from the Escobedo strain compared with the susceptible New Orleans strain.

Strain	N ^a^	α-esterases (nmol/mg ptn/min)	b-esterases (nmol/mg ptn/min)	P450 (μg Cyt/mg ptn)	GST (nmol/mg ptn/min)
		Mean+SD ^c^	Mean+SD	Mean+SD	Mean+SD
L arvae					
New Orleans ^b^	30	1.57 ± 0.21	13.64± 0.83	23.30 ± 1.82	0.015 ± 0.001
Escobedo	30	2.22 ± 0.37 ***	14.56 ± 1.50 *	24.13 ± 2.22 ns	0.031 ± 0.003 ***
Adults					
New Orleans ^d^	30	1.16 ± 0.15	11.20 ± 1.32	29.32 ± 4.00	0.032 ± 0.01
Escobedo	30	1.45 ± 0.07 ***	15.79 ± 0.92 ***	35.98 ± 4.47 ***	0.039 ± 0.009 **

^a^ Sample size, ^b^ Susceptible reference strain larvae, ^c^ Mean value for each enzymatic activity ± Standard deviation, ^d^ Susceptible reference strain females. Statistical significance with respect to the susceptible strain (NO) (Mann–Whitney U test): ns = Not significant (*p* > 0.05), * *p* ≤ 0.05, ** *p* ≤ 0.01, *** *p* ≤ 0.001.

**Table 4:** Lethal concentrations (LC_50_ and LC_90_) in µg mL^−1^ for *Aedes aegypti* larvae exposed to *Salvia munzii* essential oil.

Strain/Population	N	LC_50_ (95% CI)	LC_90_ (95% CI)	Slope ± SE	χ^2^ (df)	RR_50_
New Orleans	660	184.38 (171.43–198.15)	282.28 (254.88–328.02)	6.93 ± 0.52	26.22 (9)	1
Escobedo	600	305.04 (289.23–321.17)	516.06 (479.14–565.39)	5.61 ± 0.39	8.28 (8)	1.65

N, number of larvae tested. CI, confidence intervals. SE, standard error. χ^2^ values correspond to the goodness-of-fit test of the probit model with the indicated degrees of freedom (df). RR, resistance ratio calculated as LC_50_ of the field population divided by LC_50_ of the susceptible New Orleans strain.

**Table 5:** Oviposition deterrent activity of *Salvia munzii* essential oil against two *Aedes aegypti* strains (New Orleans and Escobedo) under laboratory conditions expressed as oviposition activity index (OAI) values and the estimated RC_50_ (µg mL^−1^) obtained by Probit analysis.

Strain	Concentration (µg mL^−1^)	OAI	RC_50_ (µg mL^−1^) (95% CI)	Slope ± SE	χ^2^ (df)
New Orleans	0.1	−0.005	1.65 (1.13–2.41)	1.48 ± 0.083	38.48 (8)
	0.3	−0.049			
	0.5	−0.160			
	0.8	−0.184			
	1.0	−0.349			
	2.0	−0.393			
	4.0	−0.455			
	7.0	−0.541			
	10.0	−0.787			
	15.0	−1.000			
Escobedo	0.1	−0.004	1.73 (1.02–2.88)	1.56 ± 0.088	36.42 (6)
	0.3	−0.099			
	0.5	−0.138			
	1.0	−0.209			
	4.0	−0.360			
	7.0	−0.644			
	10.0	−0.965			
	15.0	−1.000			

OAI, oviposition activity index calculated as (NT − NC)/(NT + NC), where NT is the number of eggs laid in the treated ovitrap and NC is the number of eggs laid in the control ovitrap. OAI values range from −1 to +1; negative values indicate oviposition deterrence and positive values indicate attraction. RC_50_ represents the concentration required to reduce ovipositionby 50%. CI, confidence intervals. SE, standard error. χ^2^ values correspond to the goodness-of-fit of the Probit model with the indicated degrees of freedom (df).

**Table 6:** Oviposition deterrent activity of *Salvia munzii* essential oil against the *Aedes aegypti* Escobedo strain under semi-field conditions expressed as oviposition activity index (OAI) values and the estimated RC_50_ (µg mL^−1^) obtained by Probit analysis.

Concentration (µg mL^−1^)	OAI	RC_50_ (µg mL^−1^) (95% CI)	Slope ± SE	χ^2^ (df)
0.5	−0.313	0.62 (0.31–0.98)	1.35 **±** 0.16	2.13 (2)
2.0	−0.556			
10.0	−0.902			
40.0	−1.000			

OAI, oviposition activity index calculated as (NT − NC)/(NT + NC), where NT is the number of eggs laid in the treated ovitrap and NC is the number of eggs laid in the control ovitrap. OAI values range from −1 to +1; negative values indicate oviposition deterrence and positive values indicate attraction. RC_50_ represents the concentration required to reduce oviposition by 50%. CI, confidence intervals. SE, standard error. χ^2^ values correspond to the goodness-of-fit of the Probit model with the indicated degrees of freedom (df).

## Data Availability

The original contributions presented in the study are included in the article/Supplementary Materials. Further inquiries can be directed to the corresponding author.

## Supplementary Materials

The following supporting information can be downloaded at: https://www.mdpi.com/article/10.3390/tropicalmed11050134/s1. Table S1: Raw egg counts used to calculate the oviposition activity index (OAI) for the susceptible New Orleans strain and the resistant Escobedo strain of *Aedes aegypti* under laboratory conditions. Table S2: Raw data used to calculate the median repellent concentration (RC_50_) for the susceptible New Orleans strain and the resistant Escobedo strain of *Aedes aegypti* under laboratory conditions. Table S3: Raw egg counts used to calculate the oviposition activity index (OAI) for persistence assays in the susceptible New Orleans strain and the resistant Escobedo strain of *Aedes aegypti* under laboratory conditions. Table S4: Raw egg counts used to calculate the oviposition activity index (OAI) for the resistant Escobedo strain of *Aedes aegypti* under semi-field conditions. Table S5: Raw data used to calculate the median repellent concentration (RC_50_) for the resistant Escobedo strain of *Aedes aegypti* under semi-field conditions. Figure S1: GC–MS chromatogram of the *Salvia munzii* essential oil used in the present study.
