# Supplementary material for: Oviposition Deterrent and Larvicidal Activity of Salvia munzii Essential Oil Against Susceptible and Insecticide-Resistant Aedes aegypti
Source: Trop Med Infect Dis. 2026 May 15;11(5):134. doi: 10.3390/tropicalmed11050134 (PMC13211698; doi:10.3390/tropicalmed11050134)
Supplement: Supplementary file 1 [file tropicalmed-11-00134-s001.zip › Figure S1.pdf]

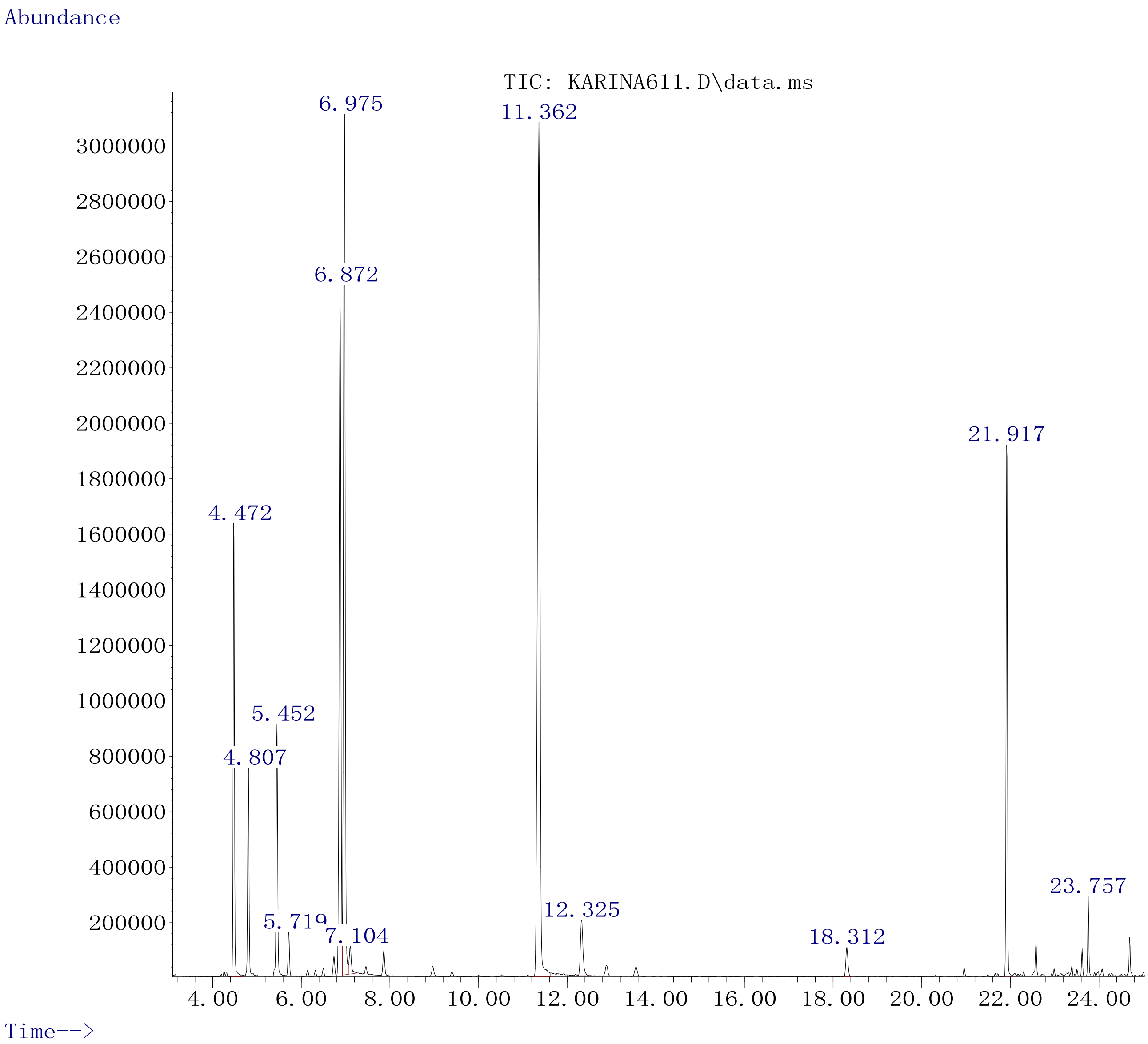

Figure S1. GC–MS chromatogram of the *Salvia munzii* essential oil used in the present study.
