# Supplementary material for: Oviposition Deterrent and Larvicidal Activity of Salvia munzii Essential Oil Against Susceptible and Insecticide-Resistant Aedes aegypti
Source: Trop Med Infect Dis. 2026 May 15;11(5):134. doi: 10.3390/tropicalmed11050134 (PMC13211698; doi:10.3390/tropicalmed11050134)
Supplement: Supplementary file 1 [file tropicalmed-11-00134-s001.zip › Supplementary_tables.pdf]

Table S1. Raw egg counts used to calculate the oviposition activity index (OAI) for the susceptible New Orleans strain and the resistant Escobedo strain of *Aedes aegypti* under laboratory conditions.

Strain/ Population	Concentration (μg/mL^{-1})	Control					Treatment				
		No. of eggs					No. of eggs				
		R1	R2	R3	R4	Total	R1	R2	R3	R4	Total
New Orleans	0.1	166	175	205	205	751	200	182	191	171	744
	0.3	130	124	166	176	596	136	157	146	101	540
	0.5	138	164	154	136	592	133	80	124	92	429
	0.8	121	110	121	144	496	89	94	88	71	342
	1	91	102	86	115	394	45	36	60	49	190
	2	82	82	64	70	298	38	12	37	43	130
	4	60	95	86	74	315	43	25	20	30	118
	7	105	88	70	63	326	19	32	26	20	97
	10	87	64	96	81	328	11	28	0	0	39
	15	98	78	86	85	347	0	0	0	0	0
Escobedo	0.1	200	210	152	203	765	188	173	182	216	759
	0.3	158	177	184	149	668	130	155	132	131	548
	0.5	124	115	175	143	557	115	110	98	99	422
	1	99	122	127	115	463	69	78	74	82	303
	4	127	97	79	92	395	55	42	37	52	186
	7	204	60	115	69	448	21	24	29	23	97
	10	159	101	98	87	445	5	1	1	1	8
	15	98	105	115	80	398	0	0	0	0	0

Table S2. Raw data used to calculate the median repellent concentration (RC_{50}) for the susceptible New Orleans strain and the resistant Escobedo strain of *Aedes aegypti* under laboratory conditions.

Strain/ Population	Concentration (μg/mL^{-1})	Control	Treatment	%ER (Effective Repellency)	%Total Effective Repellency
		No. of eggs	No. of eggs		
		Total	Total		
New Orleans	0.1	751	744	1	100
	0.3	596	540	9	100
	0.5	592	429	28	100
	0.8	496	342	31	100
	1	394	190	52	100
	2	298	130	57	100
	4	315	118	62	100
	7	326	97	70	100
	10	328	39	88	100
	15	347	0	100	100
Escobedo	0.1	765	759	1	100
	0.3	668	548	18	100
	0.5	557	422	24	100
	1	463	303	34	100
	4	395	186	53	100
	7	448	97	78	100

	10	445	8	95	100
	15	398	0	100	100

Table S3. Raw egg counts used to calculate the oviposition activity index (OAI) for persistence assays in the susceptible New Orleans strain and the resistant Escobedo strain of *Aedes aegypti* under laboratory conditions.

Strain/ Population	Days	RC_{50}									
		Control					Treatment				
		No. of eggs					No. of eggs				
		R1	R2	R3	R4	Total	R1	R2	R3	R4	Total
New Orleans	5	82	76	78	80	316	42	45	35	39	161
	10	78	81	83	79	321	44	49	39	42	174
	15	80	79	81	86	326	47	53	43	49	192
	20	81	77	80	76	314	49	55	47	52	203
	25	79	72	81	83	315	53	59	49	59	220
	30	83	81	77	79	320	59	61	57	63	240
	35	88	79	81	77	325	61	65	63	68	257
	40	84	76	80	75	315	67	73	69	71	280
Escobedo	5	110	115	96	121	442	59	68	63	71	261
	10	105	98	109	118	430	62	73	75	81	291
	15	99	115	120	115	449	78	79	81	77	315
	20	110	97	105	119	431	81	80	79	80	320
	25	122	98	115	103	438	88	92	90	96	366
	30	119	102	97	132	450	96	102	110	115	423
	35	102	98	115	119	434	125	118	132	141	516
	40	115	110	99	105	429	138	142	151	162	593

Strain/ Population	Days	10 $\mu g/mL^{-1}$									
		Control					Treatment				
		No. of eggs					No. of eggs				
		R1	R2	R3	R4	Total	R1	R2	R3	R4	Total
New Orleans	5	88	79	83	91	341	12	9	9	11	41
	10	79	88	81	79	327	13	10	8	9	40
	15	81	83	79	84	327	12	9	9	11	41
	20	77	82	83	79	321	15	12	10	14	51
	25	85	83	79	75	322	15	15	13	17	60
	30	91	88	83	82	344	16	12	18	15	61
	35	82	86	79	77	324	17	15	15	19	66
	40	91	88	79	73	331	18	15	17	16	66
Escobedo	5	98	89	88	96	371	1	2	2	3	8
	10	102	95	85	93	375	1	2	2	4	9
	15	99	98	89	95	381	1	2	3	3	9
	20	102	95	99	89	385	2	2	3	4	11
	25	98	95	102	91	386	3	3	3	4	13
	30	102	98	96	97	393	4	5	5	6	20
	35	95	96	101	98	390	5	7	7	8	27

	40	89	99	96	95	379	8	8	9	11	36

		15 µg/mL-1									
Strain/ Population	Days	Control					Treatment				
		No. of eggs					No. of eggs				
		R1	R2	R3	R4	Total	R1	R2	R3	R4	Total
New Orleans	5	101	88	85	83	357	0	0	0	0	0
	10	95	87	101	86	369	0	0	0	0	0
	15	100	88	79	82	349	0	0	0	0	0
	20	89	93	95	79	356	0	0	0	0	0
	25	88	93	98	89	368	0	0	0	0	0
	30	83	88	92	90	353	0	1	0	0	1
	35	88	86	79	82	335	1	1	0	0	2
	40	95	93	77	80	345	1	1	1	0	3
Escobedo	5	98	97	105	100	400	0	0	0	0	0
	10	99	89	98	97	383	0	0	0	0	0
	15	95	96	97	99	387	0	0	0	0	0
	20	101	99	95	93	388	0	0	0	0	0
	25	98	95	102	92	387	0	0	0	0	0
	30	105	98	89	96	388	0	1	0	1	2
	35	110	92	96	97	395	0	1	0	1	2
	40	105	98	99	92	394	0	1	1	1	3

Table S4. Raw egg counts used to calculate the oviposition activity index (OAI) for the resistant Escobedo strain of *Aedes aegypti* under semi-field conditions.

Strain/ Population	Concentration (µg/mL-1)	Treatment					
		No. of eggs					
		R1	R2	R3	R4	R5	Total
Escobedo	0.5	65	71	66	82	90	374
	2	45	35	39	41	44	204
	10	11	5	7	8	6	37
	40	0	0	0	0	0	0
	Control	160	155	117	144	139	715

Table S5. Raw data used to calculate the median repellent concentration (RC_{50}) for the resistant Escobedo strain of *Aedes aegypti* under semi-field conditions.

Strain/ Population	Concentration (µg/mL-1)	Treatment No. of eggs Total	%ER (Effective Repellency)	%Total Effective Repellency
Escobedo	0.5	374	48	100
	2	204	71	100
	10	37	95	100
	40	0	100	100
	Control	715	-	-
